# Faster rhythmic auditory stimulation induced less severe movement abnormalities in people with psychotic-like experiences

**DOI:** 10.1192/j.eurpsy.2025.879

**Published:** 2025-08-26

**Authors:** S.-M. Wang, L.-C. Kuo, H.-M. Hsu

**Affiliations:** 1 Department of Long-Term Care, National Taipei University of Nursing and Health Sciences, Taipei; 2 Department of Occupational Therapy; 3 Medical Device Innovation Center; 4 Institute of Allied Health Sciences, National Cheng Kung University, Tainan; 5 Point Robotics Medtech Incorporation, Taipei, Taiwan, Province of China

## Abstract

**Introduction:**

People with psychotic-like experiences (PLE) have slow movements and uncontrolled movements, which are indicative of transition to psychotic disorders afterwards. Earlier research has reported that rhythmic auditory stimulation (RAS) is a promising therapeutic technique for movement abnormalities in people in the psychosis continuum. However, the small sample size was a major limitation in earlier research and restricted result generalizability.

**Objectives:**

This study was to increase the sample size and examine if faster RAS induced faster movements and less uncontrolled movements at both hands in people with PLE.

**Methods:**

A total of 55 right-handed people with PLE (age: 20.51±2.50 years; 28 females) and 55 age- and gender-matched right-handed healthy controls (age: 20.53±3.10 years; 24 females) were recruited. Participants used the index finger to perform the alternate touching task for each hand when the motion capture system recorded the movement procedure. They were required to follow each beat of RAS with the normal tempo (100% of the fastest movement tempo without RAS) and the fast tempo (110% of the fastest movement tempo), the order of which was counterbalanced, when performing the alternate touching task. Kinematic variables were calculated to reflect severity of slow movements and uncontrolled movements in participants.

**Results:**

Two-way analysis of variance showed no interaction between groups and RAS in right-hand and left-hand kinematic values. People with PLE had slow movements at both hands and uncontrolled movements at the right hand. Faster RAS induced faster movements and less uncontrolled movements at both hands in people with PLE.

**Conclusions:**

The major contribution of this study was to use a relatively large sample size to demonstrate effectiveness of faster RAS on inducing faster movements and less uncontrolled movements at both hands in people with PLE and thus increase result generalizability. Given that movement abnormalities are initial signs in the psychosis continuum and risk factors of transition to psychotic disorders, when healthcare practitioners design early intervention for movement problems in people with PLE, incorporating RAS in therapy is suggested.

**Disclosure of Interest:**

None Declared

